# Symptom Attribution, Help Seeking and Willingness to Undergo Diagnostic Investigations for Bowel Cancer Symptoms in People With Anxiety and/or Depression—A Vignette Study

**DOI:** 10.1002/pon.70151

**Published:** 2025-04-12

**Authors:** Bettina Friedrich, Cristina Renzi, Lucy Mitchinson, Rupert A. Payne, Samuel W. D. Merriel, Georgios Lyratzopoulos, Gary Abel, Christian von Wagner

**Affiliations:** ^1^ Research Department of Behavioural Science and Health University College London London UK; ^2^ Faculty of Medicine University Vita‐Salute San Raffaele Milan Italy; ^3^ Centre for Prevention, Detection and Diagnosis Queen Mary University of London London UK; ^4^ Health and Community Sciences University of Exeter Exeter UK; ^5^ Centre for Primary Care and Health Services Research University of Manchester Manchester UK

**Keywords:** attribution, bowel cancer, cancer, comorbidity, diagnostic test, early detection, health psychology, help seeking, mental health, oncology

## Abstract

**Background:**

Bowel cancer is a common cause of cancer deaths and survival increases dramatically with earlier diagnosis. People with mental health problems such as anxiety and/or depression (A/D) are less likely to engage in health behaviours important for early detection.

**Aims:**

We explored whether three processes crucial for early cancer diagnosis are different in the A/D group compared to controls: (1) ‘attributions’ that is, assumed causes of bodily changes typical for bowel cancer, (2) help seeking actions participants would be likely to take and (3) willingness to do a relevant test (stool test or sigmoidoscopy/colonoscopy).

**Methods:**

In this randomised online vignette study, 1883 participants were presented one of four scenarios that each featured one bodily change typical of bowel cancer. Attributions were indicated using a free text response option. Help seeking options were presented in a list and likelihood of engaging in them was explored. Willingness to take a test was assessed using a question with response options ‘yes’, ‘no’ and ‘unsure’.

**Results:**

Participants with A/D were more likely to attribute symptoms to their mental health than to cancer. Male participants with A/D were less likely to engage in several help‐seeking actions. Participants in the A/D group were less likely to be willing to undergo invasive diagnostic testing.

**Conclusions:**

People with A/D should be encouraged to take bodily changes typical of cancer seriously and be proactive in help seeking and taking tests. Clinicians need to be aware that people with A/D may misattribute bodily changes associated with cancer and support them to seek help and undergo testing.

## 1 Background

Bowel cancer is the second most common cause of cancer deaths in the UK, accounting for almost 17,000 deaths per year between 2017 and 2019 [1]. More than 9 in 10 people with bowel cancer survive their disease for 5 years or more if diagnosed at the earliest stage, but survival rates drop dramatically with later stage diagnoses [2]. Early diagnosis of cancer is strongly linked with people's willingness and ability to engage in screening programmes, recognise symptoms, seek help, and undergo diagnostic testing [3, 4]. The ease of which people can engage with the care system and benefit from earlier cancer diagnosis varies for different populations. People with pre‐existing comorbidities (who make up about 50% of the UK population) can face specific challenges to early cancer diagnosis as their conditions may conceal or suspend investigation of a potential cancer [5].

One group of conditions which is associated with poorer cancer outcomes are people living with long term mental health morbidity (MHM) [6]. More specifically, people living with MHM have been found to be less likely or unable to engage in important health behaviours, such as leading healthy lifestyles or treatment adherence [7, 8] and more likely to report negative experiences when seeking care [9, 10] and to experience stigma by health care professionals [11].

The most recent evidence comes from a vignette study [12], in which symptom attribution and intended help‐seeking for bowel cancer related symptoms among people with and without Type 2 diabetes were compared. A subgroup analysis of 183 adults living with MHM and 1104 adults without MHM revealed that there was no difference in symptom attribution. Respondents with MHM were, however, significantly less likely to contact a GP if they experienced a change in bowel habit or mention rectal bleeding. Moreover, respondents living with MHM were less willing to undergo a colonoscopy to investigate the cause of their symptoms. However, as the primary aim of the study did not target MHM, it only partly addressed potential differences in symptom attribution, intended help seeking, and attitudes towards diagnostic testing.

The present study aimed to provide a more in‐depth investigation of differences between people with self‐reported anxiety and/or depression, and those without such conditions, in symptom attribution, help‐seeking, and willingness to undergo diagnostic testing, if they were to experience potential colorectal cancer symptoms. Anxiety and depression are among the most common types of MHM in the UK and worldwide [13, 14]. Both conditions have been associated with delayed cancer diagnosis in a study investigating the time to diagnosis in bowel cancer [15, 16]. Additionally, anxiety and depression were associated with 2‐fold longer diagnostic intervals for bowel cancer and a higher likelihood of emergency diagnosis [17]. Less is known about how anxiety and depression influence patient symptom attribution and help‐seeking. Burgess and colleagues [18] reported no association between patient delay and prevalence of depression or anxiety in the year preceding symptom discovery. However, other studies suggest that anxiety and depression are [19] associated with longer help‐seeking intervals for colorectal cancer symptoms. To further explore the role of anxiety and depression on symptom attribution, help seeking, and willingness to undergo diagnostic testing for bowel cancer related symptoms, we purposefully recruited individuals living with self‐reported anxiety and/or depression and those without for a vignette study.

## 2 Methods

### 2.1 Study Design and Participants

The study was developed with a Public and Patient Involvement (PPI) panel of people with lived experience of cancer. Some panel members had also lived experience with A/D. The panel influenced the design of the study and reflected on the findings with the research team.

We developed four vignette scenarios each featuring a possible colorectal cancer symptom: (1) rectal bleeding, (2) abdominal pain, (3) fatigue or (4) changes in bowel habit (see Table A1). All participants were randomly assigned to one of the four scenarios. The vignettes and study questions were hosted in an online survey on Qualtrics software, Copyright 2023 (www.qualtrics.com). Table A2 shows the distribution of participants across the different vignettes.

Participants were recruited using the Prolific online recruiting platform for scientific research and commercial marketing studies (www.prolific.com). Participants were part of an online panel with 57,620 active users in the UK. Prolific collects self‐reported baseline data on demographic characteristic, including the presence of health conditions based on a ‘yes’ or ‘no’ response to a pre‐specified list of conditions.

Eligibility criteria were (1) people in the UK, (2) aged 50 years and over and (3) who had not reported cancer or psychosis. The age restriction reflects the population who experience greater cancer risk. People with a previous cancer diagnosis were excluded as they may be primed to attribute symptoms to cancer. People with psychosis were excluded as the nature of this comorbidity is very different to anxiety and depression, and the issues around health behaviour when experiencing cancer symptoms are likely to be very distinct [11, 20]. We believe therefore that it is important to investigate psychosis as a preexisting condition for the diagnosing of cancer separately, and we are focusing here on an A/D population (and controls) that do not have psychosis.

We used the Prolific‐provided data to advertise the study to specific individuals that fitted our inclusion criteria and implemented quota sampling to have an equal number of A/D and controls (about 1000 in each group). Further questions in the survey were then used to remove those with a previous cancer or psychotic disorder diagnosis, and to double‐check that they met all inclusion criteria.

Patients who selected options for having ‘Depression’ or ‘Anxiety or Generalised Anxiety Disorder’ (or both) in survey responses were grouped together into a single anxiety and/or depression group (A/D group) because of a high co‐occurrence of the two conditions. The information regarding A/D was self‐reported and independent from the recency of a diagnosis. The other participants (i.e., those not having A/D) formed the control group. Presence of A/D was our key exposure of interest.

The target sample of 2000 participants was chosen based on previous vignette studies [21] and estimated to be adequate for detecting a 10% difference (80% power, *p* < 0.05) in anticipated help‐seeking between participants with and without a mental health morbidity.

Data were collected between July and August 2023. The recommendations of the Prolific platform for a compensation of £2 for 20‐min survey (equivalent to £6/h) for participants were followed. Ethical approval was granted by the University College London Ethics Committee (ref: 16891.003). Participants were not provided any information about the aim of the study, so they would not be primed about the topic ‘cancer’ before responding. We collected informed consent at the very beginning of the survey and only participants who gave consent were included in this study.

### 2.2 Covariates

We used age and gender of participants as covariates for calculations. Furthermore, we collected data on mental and physical health. Based on these data, we defined a binary variable indicating the presence of a physical health condition, and a three‐level variable indicating the presence of no, one, or two or more neurodivergent or mental health conditions (not including A/D): obsessive compulsive disorder (OCD), bi‐polar disorder, post‐traumatic stress disorder (PTSD), another long‐term mental health condition, autism or autism spectrum condition, or learning disability. Participants were also asked whether they had previously had a colonoscopy/sigmoidoscopy or completed a home‐based stool test for bowel cancer.

### 2.3 Outcome Variables

The item text used to explore the three outcome variables are presented in Table A3.

#### 2.3.1 Symptom Attribution

Participants indicated in a free text format what they thought the possible cause for the symptoms described in the vignettes could be. There was no limitation regarding the numbers of causes they could mention.

#### 2.3.2 Intended Help Seeking

Participants indicated on a four‐point Likert scale (‘definitively would’—to ‘definitively would not’) whether they would be willing to take each of the 14 help seeking behaviours presented in a list if they experience the bodily change. They could also reply ‘not applicable’.

#### 2.3.3 Willingness to Undergo Testing

Finally, respondents indicated if they would be willing to have a stool test or a sigmoidoscopy/colonoscopy if they experienced symptoms described in the vignettes, and if a doctor recommended such tests. A stool test was proposed to participants whose vignette featured ‘change in bowel habit’ or ‘fatigue’. In contrast we proposed sigmoidoscopy/colonoscopy to those whose vignette mentioned ‘rectal bleeding’ or ‘abdominal pain’.

### 2.4 Statistical Analysis

Data analyses were conducted using Stata version 18 (StataCorp LLC, College Station, TX).

#### 2.4.1 Symptom Attribution

A content analysis of the free text responses for the symptom attribution was conducted. First all responses were listed and then meaningful categories such as ‘cancer’ or ‘mental health’ were developed into which the responses were then grouped. We then ranked these categories according to the percentage of participants mentioning them—separately for A/D and controls. For each of the six most frequently mentioned attribution categories overall, logistic regressions were used to model the association between A/D and mentioning a given attribution category. We controlled for age, gender, number of neurodiversity/mental health conditions and whether they have a physical health condition. We also included an interaction term between gender and having A/D for attributions.

#### 2.4.2 Intended Help Seeking

The four‐point Likert scale responses were dichotomised to ‘would take behaviour’ or ‘would not take behaviour’. Logistic regression examined the associations between anxiety/depression and individual intended actions. As preliminary regressions showed strong differences interaction effects for gender and A/D (measured through a respective interaction variable), we subsequently calculated regression models for all individual action items separately by gender. Again, we included age, number of physical comorbidities and whether they had any other neurodiversity comorbidity as potential confounding variables.

#### 2.4.3 Willingness to Engage in Diagnostic Tests

We used logistic regression analysis to examine the association between anxiety and/or depression and willingness to have a stool test or a colonoscopy/sigmoidoscopy (if they experienced the featured bodily change) in two separate regression models. Again, we included age, gender, number of other mental health/neurodiversity conditions and whether they had physical comorbidities as control variables. Furthermore, we controlled whether they had done a respective test before or not. We also included an interaction term between gender and having anxiety and/or depression for intentions to have follow up testing.

Interactions between gender and A/D were explored for all regression models. These were significant in the models examining help seeking, so we subsequently performed models stratified by gender.

## 3 Results

### 3.1 Demographics

Among the 2022 individuals consenting to take part in the study, 111 were excluded as they did not meet the inclusion criteria (i.e., history of cancer *n* = 95, psychotic disorders *n* = 8, aged < 50 years *n* = 8) and 28 were excluded for not completing the vignette questions. This resulted in the inclusion of 1883 participants (93% of those who consented). Our quota sampling method ensured that we had a similar number of people with and without A/D for each vignette (see Table A2).

Table 1 shows the breakdown of participant characteristics. The majority of participants were white (95%), female (63%), and within the youngest age category (64% aged 50–59). The sample ranged in numbers of comorbidities with 20% reporting no additional conditions (excluding anxiety/depression) and 24% reporting ‘four or more comorbidities’ (not including anxiety or depression). The most frequently reported conditions were being overweight or obese (39.3% of total sample), arthritis (27.6%), high blood pressure (24%), and long‐term back problems (23.3%). Table A4 provides an overview of the frequency of long‐term health conditions experienced by participants with and without A/D.

**TABLE 1:** Characteristics of the overall sample, anxiety and/or depression group, and control group.

Variable	Total	Anxiety and/or depression group	Control group	*χ* ^2^ *p* value
	*N* (col %)	*N* (col %)	*N* (col %)	
Total	1883	901 (47.8)	982 (52.2)	
Gender				
Male	681 (36.2)	265 (29.4)	416 (42.4)	*p* < 0.001
Female	1189 (63.1)	628 (69.7)	561 (57.1)	
Self‐describe/PNTS	13 (0.7)	8 (0.9)	5 (0.5)	
Age				
50–59	1208 (64.1)	652 (72.4)	556 (56.5)	*p* < 0.001
60–69	555 (29.5)	210 (23.3)	345 (35.1)	
70–79	112 (6)	38 (4.2)	74 (7.5)	
80+	8 (0.4)	1 (0.1)	7 (0.7)	
Ethnicity				
White	1792 (95.2)	866 (96.1)	926 (94.3)	*p* = 0.18
BAME	80 (4.3)	31 (3.4)	49 (5)	
Other/PNTS	11 (0.6)	4 (0.4)	7 (0.7)	
Physical or mental health conditions (excluding anxiety and/or depression)				
None	378 (20.1)	65 (7.2)	313 (31.9)	*p* < 0.001
1	419 (22.3)	149 (16.5)	270 (27.5)	
2	347 (18.4)	165 (18.3)	182 (18.5)	
3	287 (15.2)	169 (18.8)	118 (12)	
4+	452 (24)	353 (39.2)	99 (10.1)	
Health management				
Good	837 (53.3)	368 (40.8)	469 (70.1)	*p* < 0.001
Average	602 (38.3)	429 (47.6)	173 (25.9)	
Bad	130 (8.3)	103 (11.4)	27 (4)	
PNTS	1 (0.1)	1 (0.1)	0	
Contact with HCP				
Once a year or more	1032 (65.7)	644 (71.5)	388 (58)	*p* < 0.001
Less than once a year	535 (34.1)	255 (28.3)	280 (41.9)	
PNTS	3 (0.2)	2 (0.2)	1 (0.2)	
Average GP visits in the last year				
None	454 (24.9)	143 (16.4)	311 (32.7)	*p* < 0.001
Once	391 (21.4)	150 (17.2)	241 (25.3)	
Between 1 and 6	772 (42.3)	411 (47)	361 (38)	
Between 6 and 12	164 (9)	130 (14.9)	34 (3.6)	
12+	44 (2.4)	40 (4.6)	4 (0.4)	
Average A&E visits last year				
None	1533 (83.7)	691 (79)	842 (88.1)	*p* < 0.001
1	210 (11.5)	116 (13.3)	94 (9.8)	
2	64 (3.5)	46 (5.3)	18 (1.9)	
3	24 (1.3)	22 (2.5)	2 (0.2)	
Screening history (‘have you ever engaged in a screening test for cancer?’)				
Yes	1312 (70.9)	634 (71.4)	678 (70.4)	*p* = 0.78
No	504 (27.2)	236 (26.6)	268 (27.8)	
Unsure/PNTS	35 (1.9)	18 (2)	17 (1.8)	

*Note:* χ^2^ tests were used to compare participants with anxiety and/or depression to those without.

Of our 1883 participants, 901 (47.8%) reported having A/D. Of these, 511 (56.7%) reported having both conditions, 198 (22%) reported having anxiety only, and 192 (21.3%) reported having depression only. The majority reported having received a diagnosis for their mental health condition over 5 years ago (71.4% of those with anxiety and 79.4% of those with depression). When considering the severity of their mental health conditions, most participants described their condition to be ‘overall’ moderate (61.3%). Over one fifth felt that their mental health condition was severe (22.8%) and 15.6% described it as minor. When asked about how their mental health was ‘right now’, half of the participants felt their mental health condition was currently of moderate (49.7%) or minor severity (42.7%). The most common treatment for depression was anti‐depressant medication (reported by 58.3% of participants with depression only and 61.6% of participants with anxiety and depression) (Table A5).

Participants in the A/D group were more often female (69.7% vs. 57% control), in the younger age group of 50–59 (72.4% A/D vs. 56.5% control), most often reported having four or more other co‐morbidities (39.2% vs. 10.1% control) and worse management of health (40.8% of the A/D group described their health management as good compared to 70.1% of control). Participants in the A/D group also reported a greater level of contact with healthcare professionals, GPs, and emergency services, than the control group (see Table 1).

### 3.2 Symptom Attribution

As Table 2 shows, people in the A/D group would attribute the symptoms to their underlying mental health condition or cancer with similar frequency (28.6% and 28.1%, respectively). Among the control group, a higher proportion of respondents would attribute symptoms to cancer (34.3%) than a mental health condition (14.2%). Cancer was the condition most often attributed to by participants in the control group (rank 1), compared to being the fourth rank condition for participants in the A/D group.

**TABLE 2:** Ranking of attributions.

Attribution	Rank		% (*N*)	
	Control group	Anx/dep	Control group (total = 982)	Anx/dep (total = 901)
Cancer	1	4	34.3 (337)	28.1 (253)
Diet	2	2	31.9 (313)	32.2 (290)
Haemorrhoids	3	5	16.3 (160)	17.3 (156)
Mental health	4	3	14.2 (139)	28.6 (258)
Indigestion	5	1	13.2 (130)	44 (396)
Bug infection	6	8	12.3 (121)	10.4 (94)
Irritable bowel syndrome (IBS)	7	6	12.1 (119)	16.3 (147)
Lifestyle	8	9	10 (98)	9.1 (82)
Sleep	9	7	9.7 (95)	11.2 (101)
Constipation	10	11	9.1 (89)	6.4 (58)
Unclear	11	14	7.3 (72)	4.8 (43)
Age/menopause	11	17	6.3 (62)	3.8 (34)
Injury tear	13	12	5.8 (57)	5.5 (50)
Non specified/rare	14	10	5.4 (53)	7.4 (67)
Gas	15	13	4.8 (47)	5.2 (47)
Stomach	16	16	4.1 (40)	4.4 (40)
Anaemia	16	19	4.1 (40)	2.1 (19)
Period	18	21	2.3 (23)	1.7 (15)
Meds	19	15	2.2 (22)	4.7 (42)
Muscle	20	20	2 (20)	2 (18)
Appendix	21	24	1.8 (18)	1.1 (10)
Polyps	22	22	1.7 (17)	1.3 (12)
Diabetes	23	23	1.6 (16)	1.2 (11)
Diverticulitis disease	24	18	1.4 (14)	3.2 (29)
Thyroid	25	27	1.1 (11)	0.3 (3)
Crohn's disease	26	25	0.9 (9)	1 (9)
Diarrhoea	27	29	0.8 (8)	0.2 (2)
Hernia	28	26	0.6 (6)	0.4 (4)
Growth or blockage	29	27	0.5 (5)	0.3 (3)
Chronic fatigue	30	30	0.3 (3)	0.1 (1)
Weight	30	30	0.3 (3)	0.1 (1)

**FIGURE 1:**
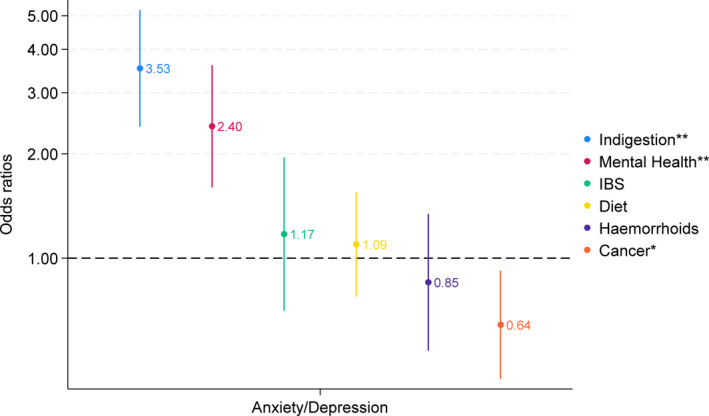
Adjusted odds ratios for attributions of people with anxiety and/or depression (reference group: controls).

As Figure 1 shows, participants in the A/D group were more likely to attribute the symptoms to mental health conditions (adjusted OR = 2.4, 95% CI 1.60–3.60. *p* < 0.001) or indigestion (adjusted OR = 3.53, 95% CI 2.39–5.20, *p* = 0.001) and less likely to attribute them to cancer (adjusted OR = 0.64, 95% CI 0.45–0.92, *p* = 0.016) than controls.

### 3.3 Intended Actions

Table 3 shows the frequency ranking for intended actions in the hypothetical scenario—for both people with anxiety and/or depression and controls. The preferences for actions are almost identical for both groups, however participants with A/D seemed generally less likely to engage in actions as the relative frequency values show. Interestingly, most participants (over 80% in both the A/D and the control group) would dismiss the symptom as something to not worry about if they experienced it. The second most common action would be to mention the symptom to the GP if they saw them for another reason. Only around 40% of participants would be willing to contact a GP specifically about the symptom.

**TABLE 3:** Ranking for help seeking behaviour.

Actions	Rank		% (*N*)	
	Controls	Anx/dep	Controls (total = 982)	Anx/dep (total = 901)
Dismiss [symptom] as something not to worry about	1	1	82.99 (815)	85.57 (771)
Mention [symptom] if you saw the GP for another reason	2	2	75.46 (741)	70.48 (635)
Wait and see what happens (e.g., if [symptom] gets worse)	3	3	71.69 (704)	69.48 (626)
Contact a diabetic specialist	4	4	58.35 (573)	55.05 (496)
Talk to members of your family about [symptom]	5	5	51.93 (510)	47.17 (425)
Contact a nurse about [symptom]	6	6	44.91 (441)	45.28 (408)
Contact the GP about [symptom]	7	7	43.08 (423)	37.85 (341)
Go to the pharmacy (chemist) for advice	8	8	16.09 (158)	13.65 (123)
Mention [symptom] if you saw a nurse for another reason	9	9	10.18 (100)	8.44 (76)
Go to A&E	10	10	9.88 (97)	6.77 (61)
Contact 111	11	11	5.3 (52)	5.44 (49)
Look up information about [symptom] online or on the NHS app	12	13	2.24 (12)	1.44 (13)
Contact an endocrinologist	13	12	1.63 (16)	1.55 (14)
Other, please specify	14	13	1.12 (11)	1.33 (12)

#### 3.3.1 Logistic Regressions

There was no significant difference between A/D and the control group for female respondents. In contrast, males in the A/D group would be significantly less likely to contact the GP (adjusted OR = 0.52, 95% CI 0.36–0.75, *p* < 0.001) or mention the bodily change, if they saw their GP for something else (adjusted OR = 0.63, 95% CI 0.41–0.98, *p* = 0.04) compare with males in the control group. They would also be less likely to go to the pharmacy (chemist) for advice (adjusted OR = 0.51, 95% CI 0.31–0.84, *p* = 0.01), go to A&E (adjusted OR = 0.44, 95% CI 0.24–0.78, *p* = 0.005), wait and see what happens (adjusted OR = 0.66, 95% CI 0.45–0.97, *p* = 0.03) or talk to a family member (adjusted OR = 0.68, 95% CI 0.48–0.97, *p* = 0.03).

### 3.4 Willingness to Have Testing

The logistic regressions showed that anxiety/depression was not significantly associated with the likelihood to do a stool test in these hypothetical scenarios.

Having anxiety and/or depression, however, significantly predicted willingness to have a colonoscopy/flexible sigmoidoscopy in the abdominal pain and rectal bleeding scenarios, with people in the A/D group being less willing to do such a test (adjusted OR = 0.57, 95% CI 0.41–0.80, *p* = 0.001).

## 4 Discussion

The aim of our study was to investigate symptom attribution, help seeking behaviour, and willingness to do a stool test or colonoscopy, in response to a hypothetical cancer‐relevant symptom among people with and without anxiety and/or depression.

People in the A/D group were more likely to attribute symptoms displayed in our vignettes to mental health problems and less likely to attribute them to cancer when compared to controls. This potential ‘downplaying’ of symptoms is problematic for early cancer diagnosis. Interestingly this finding is contrary to findings in a similar study by Pennisi et al., who did not find a difference between people with mental health problems and controls with regard to symptom attribution [19]. Pennisi's study however was secondary analysis in a study not designed to detect differences between participants that had mental health problems and those that did not—they were also lacking the power to detect a difference, which might explain the difference in findings and control. Our study does provide additional evidence for the ‘alternative explanation’ hypothesis, in that participants attributed cancer related symptoms to their mental health.

Furthermore, participants in the A/D group were less likely to engage in several help‐seeking behaviours, which is in line with the findings of Katon [7] and DiMatteo et al. [8].

Mahalik and Di Bianca argue that men might perceive help seeking for depression as a threat to their masculinity [22]. Given the strong attributions of the symptoms to mental health related issues, men would perhaps not want to draw attention to their anxiety and/or depression by seeking further help.

There was no effect of having anxiety and/or depression on the willingness to do a non‐invasive test such as stool test. On the other hand, having anxiety and/or depression made participants less likely to agree to having a more invasive colonoscopy/sigmoidoscopy. Fear of the outcomes of bowel cancer screening has been a barrier to all screening modalities including home‐based stool tests [23] but less is known about motivation to do a test. We are wondering whether our findings regarding the impact of A/D on willingness to have a colonoscopy/sigmoidoscopy might be due to concerns about physical discomfort or harms of an invasive test.

In addition to physical discomfort, there may also be barriers relating to the requirement to attend a hospital appointment and the anticipated intimacy and potential embarrassment associated with such a procedure, as suggested by Scaglioni et al. [24] and Honein‐AbouHaidar et al. [25].

### 4.1 Implications

Our findings highlight the importance of supporting people with anxiety and depression to recognise potential cancer symptoms and to facilitate help seeking behaviours and engagement with healthcare services. However, one could also argue that our findings show that the A/D participants still place cancer as a reasonably high possibility and that it might be appropriate to not assume cancer as necessarily the most likely reason for the bodily change.

It is interesting to see that while the A/D group was less likely to engage in certain help seeking actions (which was mainly driven by male participants), the ranking of the help seeking actions was almost identical in both groups, so the prioritisation of what would be important to do does not seem to be affected by A/D.

Our findings suggest extra effort may be required to encourage people with anxiety and depression to engage in diagnostic tests, particularly when these tests are of an invasive nature. These findings are potentially relevant for developing guidelines for clinicians on how to encourage people with anxiety and/or depression to look out for cancer related symptoms and report them promptly. Furthermore, guidelines should offer advice to health care professionals on how they should react when learning about relevant bodily changes in these patients. It also highlights the particular risk of not taking cancer‐related symptoms of people with anxiety and/or depression seriously given that this group is already less likely to reach out for help when experiencing the symptoms. Future research is needed to study how seeing a patient with anxiety and/or depression affects symptom attribution and decision making among health care professionals.

Therefore, targeted interventions could be developed for people with anxiety and/or depression to tackle the reported issues. The creation of cancer symptom checklists that might prompt patients with mental health problems to report bodily changes, may facilitate dialogue with health care professionals. An example of an established checklist is the SLC‐90‐R [26], which is used to assess psychological symptoms and distress. Furthermore, developing decision aids [27] for clinicians which include considerations for patients with anxiety and depression, might be a way to help health professionals better support this patient group.

Regarding cancer awareness campaigns, there seems to be a particular need to target people with depression and anxiety to ensure they do not wrongly attribute potential symptoms of bowel cancer. Finally, our findings highlight the importance of studying cancer related symptom attribution in people with a range of mental health problems and neurological conditions.

## 5 Study Strengths and Limitations

Using vignettes allowed us to explore how participants, who were not aware that the study was about cancer, would react to symptoms typical of bowel cancer. We developed scenarios similar to real‐life situations in which the reasons for bodily changes might be ambiguous to the person experiencing it. The large number of participants and detailed information on their characteristics are additional strengths of the work.

There were, however, some limitations. We relied on participants self‐reported account regarding history of anxiety and depression (and other health conditions) as well as regarding other data and we had no means of verifying this information. Therefore we need to acknowledge the possibility that there were ‘imposter participants’ who might have reported wrong information. Furthermore, the vignette study was hypothetical, so we cannot be certain whether participants would make the same attributions and take the same help seeking and diagnostic testing actions in real life. Observational studies of people who report having anxiety and/or depression might be useful to better understand whether these findings are in line with real life behaviour. It would also be interesting to explore whether self‐stigma and anticipated stigma might discourage people with mental health problems from turning to health care professionals when experiencing symptoms.

## 6 Conclusion

In summary, people with anxiety and/or depression were more likely than controls to attribute symptoms typical of bowel cancer to their mental health and less likely to attribute them to cancer. In particular, males with anxiety and/or depression were less likely to engage in a range of help seeking activities than males without these mental health conditions. People with anxiety and/or depression were also less likely to engage in invasive diagnostic tests recommended by a medical professional. Better understanding of these perceptions and health‐related behaviours in people with mental health problems, and translation of these finding into practice (e.g., campaigning, raising awareness in health care professionals, facilitating symptom conversations) could lead to an earlier diagnosis of cancer in people with mental health problems and decrease cancer related mortality in these groups.

## Author Contributions

B.F. planning and executing analysis, interpretation, writing first draft, editing, corresponding author. C.R. study conception design, review of analysis and manuscript draft, editing. L.M. design of the study, data collection, data analysis, reviewing of manuscript draft. R.A.P. review and editing of manuscript draft. S.W.D.M. study design, review and editing of manuscript draft. G.L. study conception, study design. G.A. study conception, review of analysis, editing draft manuscript. C.v.W. study conception design, review of analysis and manuscript draft, editing.

## Appendix A

**TABLE A1:** Vignette scenarios.

Vignette 1	You notice you have had changes in your normal bowel habits (such as looser poo, pooing more often or constipation) other than this symptom you have noticed no other changes
Vignette 2	You notice that you have been feeling more tired than usual (fatigued). This tiredness has made it difficult for you to go about your everyday activities. Other than this you have noticed no other changes
Vignette 3	When you use the bathroom, you notice blood in your poo (rectal bleeding). Other than this symptom you have noticed no other changes
Vignette 4	You notice you have had a cramp‐like pain in your stomach. Other than this symptom you have noticed no other changes

**TABLE A2:** Distribution of participants across the different vignettes.

	Change in bowel habit	Rectal bleeding	Fatigue	Abdominal pain	Total
	*n* (col %)	*n* (col %)	*n* (col %)	*n* (col %)	*n* (col %)
Anxiety and/or depression group	235 (50)	213 (45.3)	227 (48.2)	226 (47.9)	901 (47.9)
Control group	235 (50)	257 (54.7)	244 (51.8)	246 (52.1)	982 (52.1)
Total	470	470	471	472	1883

**TABLE A3:** Outcome measures overview.

Outcome measures	Wording of the item	Response options
Symptom attribution	What do you think the cause of [symptom] could be?	[Open text]
	Please write in the box below as many things as come to your mind. If you cannot think of any, please write ‘don't know’ in the box	
Intended help seeking	What actions would you take if you were to experience [symptom]? Next to each option, please tick the box that best reflects how likely you are to take each action	To be rated on a Likert scale: Definitively would (1)—Definitively wouldn't (4); not applicable (5) ◦ Talk to members of your family about the change in bowel habits (1) ◦ Go to the pharmacy (chemist) for advice (2) ◦ Contact the GP about the change in bowel habits (3) ◦ Mention the change in bowel habits if you saw the GP for another reason (4) ◦ Contact 111 (14) ◦ Go to A&E (5) ◦ Look up information about change in bowel habits online or on the NHS app (6) ◦ Wait and see what happens (e.g., if the change in bowel habits gets worse) (7) ◦ Dismiss change in bowel habits as something not to worry about (8) ◦ Contact a nurse about the change in bowel habits (9) ◦ Mention the change in bowel habits if you saw a nurse for another reason (10) ◦ Contact a diabetic specialist (11) ◦ Contact an endocrinologist (12) ◦ Other, please specify (13) [free text added]
Willingness to undergo testing	Still imagining that you have been experiencing [symptom], you have now spoken to a GP and they have suggested to refer you for a colonoscopy/sigmoidoscopy. (A colonoscopy/sigmoidoscopy uses a thin flexible tube with a camera on the end to look at the bowel) OR Still imagining that you have been experiencing [symptom], you have now spoken to a GP and they have suggested to refer you for a stool test. (A stool sample involves collecting a small amount of faeces in a container at home and returning it to your GP/hospital) — Would you be willing to partake in this? (A stool test was proposed to participants whose vignette featured ‘change in bowel habit’ or ‘fatigue’. In contrast we proposed sigmoidoscopy/colonoscopy to those whose vignette mentioned ‘rectal bleeding’ or ‘abdominal pain’.)	Yes (1) No (2) Unsure (3)

**TABLE A4:** Frequency of long‐term health conditions experienced by participants with and without anxiety and/or depression.

	Anxiety and/or depression		Control group		Total	
	*n*	%	*n*	%	*n*	%
Overweight or obese	475	52.7	265	27	740	39.3
Arthritis	324	36	196	20	520	27.6
High blood pressure	256	28.4	195	19.9	451	24
Long‐term back problems	288	32	151	15.4	439	23.3
IBS	219	24.3	93	9.5	312	16.6
Another mental health condition	280	31.1	32	3.3	312	16.6
Lung conditions	176	19.5	133	13.5	309	16.4
Hemorrhoids	178	19.8	81	8.3	259	13.8
Diabetes (T1 & T2)	106	11.8	64	6.5	170	9
Learning disabilities, autism, and long‐term neurological problems	119	13.2	49	5	168	8.9
Chronic constipation, diverticular disease	107	11.9	53	5.4	160	8.5
Heart conditions	42	4.66	26	2.65	68	3.61
Physical disabilities (e.g., blindness, deafness)	38	4.2	19	1.9	57	3
Another condition (including endometriosis, kidney or liver disease etc.)	165	18.34	80	8.15	245	12.99
No conditions	65	7.2	313	31.9	378	20.1

*Note:* Conditions which had < 5% prevalence in the total sample were grouped with similar conditions.

**TABLE A5:** Frequency of treatments currently used by those with anxiety and/or depression.

Current treatments	Total	Depression and anxiety		Depression only		Anxiety only		
	*N*	%	*N*	%	*N*	(%)	*N*	(%)
Not currently receiving any treatment	218	24.2	89	17.4	50	26	79	39.9
Taking anti‐anxiety medication	281	31.2	209	40.9	11	5.7	61	30.8
Taking anti‐depressant medication	448	49.7	315	61.6	112	58.3	21	10.6
Psychological therapies (e.g., talking therapies, CBT)	87	9.7	59	11.6	7	3.7	21	10.6
Receiving alternative therapies (e.g., peer support, art therapy, complementary therapies, mindfulness)	63	7	46	9	6	3.1	11	5.6
Receiving other therapies	31	3.4	19	3.7	6	3.1	6	3
Taking life‐style measures (e.g., exercise, time off work)	124	13.8	97	19	10	5.2	17	8.6
Speaking with a GP about my mental health	265	29.4	165	32.3	47	24.5	53	26.8
Prefer not to say	1	0.1	1	0.2	0	0	0	0
Total participants	901	100	511	100	192	100	198	100
